# Supplementary figures and images for: The Double Face of miR-708: A Pan-Cancer Player with Dissociative Identity Disorder
Source: Genes (Basel). 2022 Dec 16;13(12):2375. doi: 10.3390/genes13122375 (PMC9777992; doi:10.3390/genes13122375)

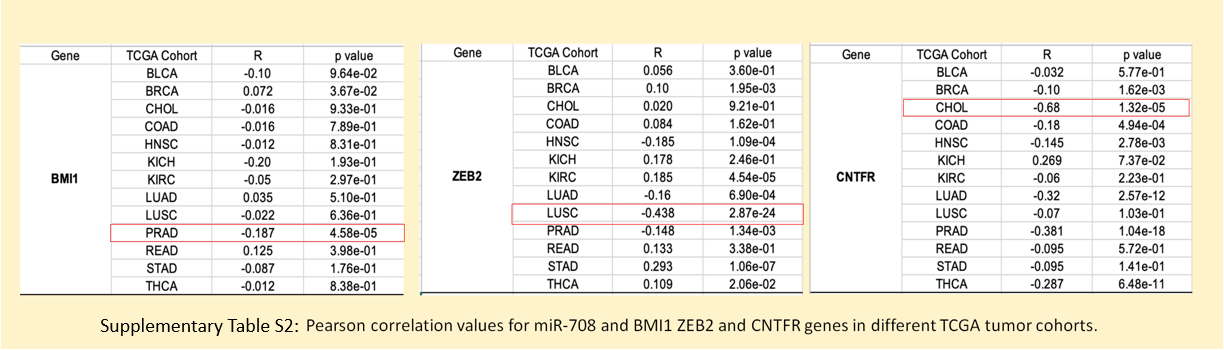

Supplement: Supplementary file 1 [file genes-13-02375-s001.zip › supplementary Table S2.png]
